# Orbital Rhabdomyosarcoma Masquerading as a Dermoid Cyst: A Case Report and Review of the Literature

**DOI:** 10.7759/cureus.50332

**Published:** 2023-12-11

**Authors:** Christina Karakosta, Maria Liaskou, Antonis Kattamis, Efthymia Rigatou, Konstantinos Paraskevopoulos

**Affiliations:** 1 Department of Ophthalmology, National and Kapodistrian University of Athens School of Medicine, Athens, GRC; 2 Department of Ophthalmology, Penteli General Hospital for Children, Athens, GRC; 3 Division of Pediatric Hematology-Oncology, First Department of Pediatrics, "Aghia Sophia" Children's Hospital, National and Kapodistrian University of Athens, Athens, GRC; 4 Department of Ophthalmology, Penteli General Hospital For Children, Athens, GRC

**Keywords:** alveolar rhabdomyosarcoma, orbital tumours, eye rhabdomyosarcoma, dermoid cysts, orbit malignancy

## Abstract

The purpose of this report is to present a case of orbital rhabdomyosarcoma (RMS) masquerading as a dermoid cyst.

A six-year-old boy with an unremarkable medical history presented in the outpatient department with a palpable mass in the superonasal region of the right orbit, which had rapidly grown in the past month. The most likely diagnosis was dermoid cyst and the patient was scheduled for surgical excision. A high index of suspicion was raised intraoperatively based on the appearance of the lesion due to the presence of a feeder vessel. The histopathology examination identified alveolar RMS. The patient was referred to a pediatric oncology department and commenced intravenous chemotherapy.

RMS may masquerade as various conditions, including dermoid cysts and chalazion. A high index of suspicion should be raised in cases with rapidly growing lesions.

## Introduction

Rhabdomyosarcoma (RMS) is a rare childhood cancer. The major anatomic sites for RMS are the head and neck region, particularly the orbit. In children, orbital RMS represents the most common primary orbital malignancy [1]. A vast majority of orbital RMS (about 90%) occur at an age younger than 16 years (the mean age of onset is five to seven years of age) [2]. Histologically, RMSs are composed of cells resembling striated muscle in various stages of embryogenesis [1]. RMS types include embryonal (the most common, accounting for approximately 60%), alveolar, pleomorphic, and spindle-cell/sclerosing types [3]. The majority of ocular rhabdomyosarcomas arise in the orbital soft tissues. In rare cases, RMS occurs in other ocular adnexal structures or even within the eye. RMS may also extend directly to the orbit from the paranasal sinuses or nasopharynx. In extremely rare cases, orbital RMS is a metastasis from distant sites [4]. Herein, a case report of masquerading orbital RMS is presented.

## Case presentation

**Figure 1:**
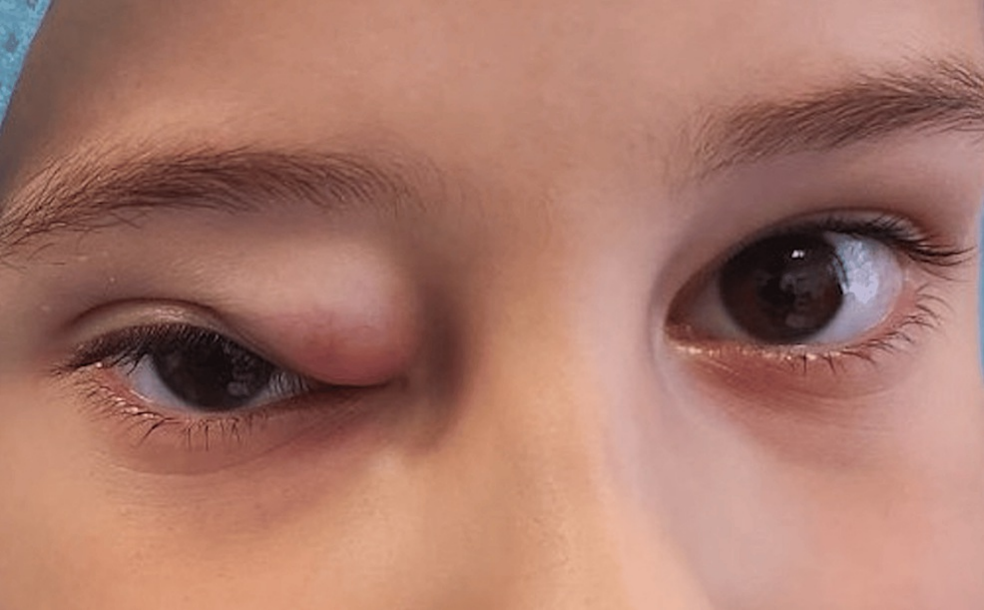
Preoperative appearance of the tumor.

A six-year-old boy with an unremarkable medical history presented in the outpatient department with a palpable mass in the superonasal region of the right orbit. The mass was firm, without pulsations, and did not expand with coughing (Figure 1). 

**Figure 2:**
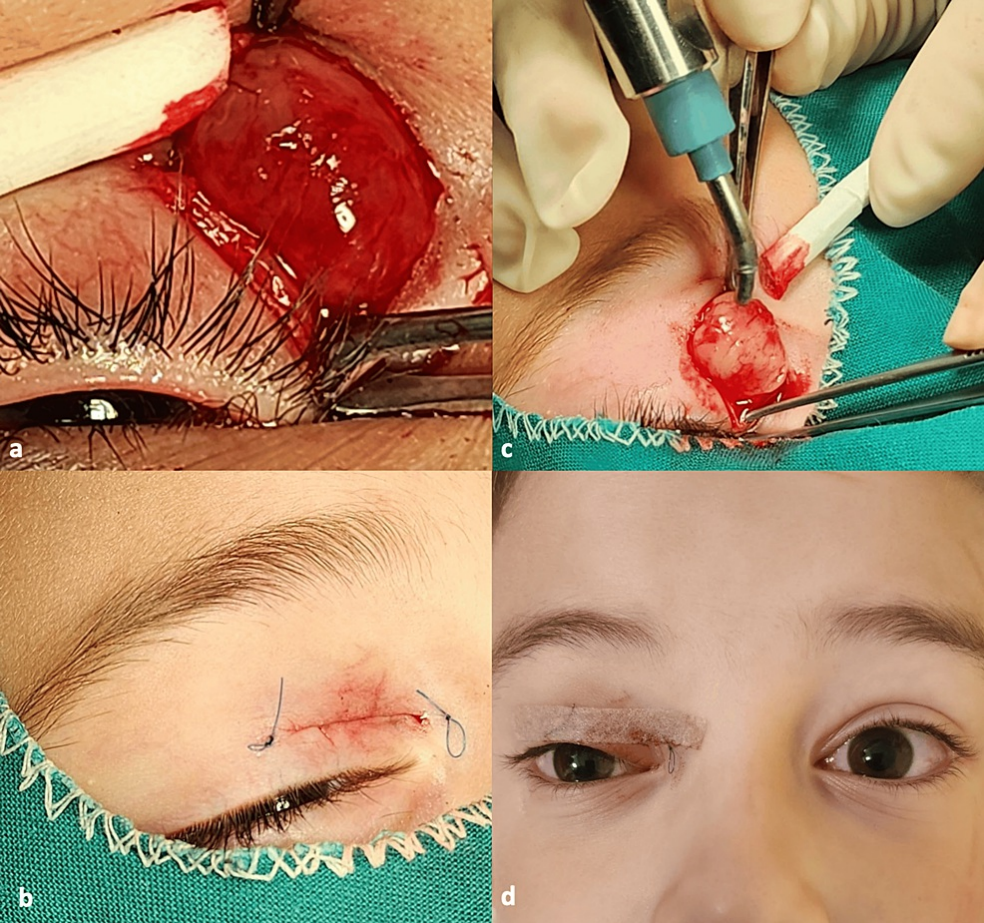
Visualization of the tumor after the incision (a), application of cryopexy to promote detachment from the surrounding tissues (b), closure of the incision (c), and postoperative appearance (d).

The patient and his parents had not noticed the presence of any symptoms, including fever. On examination, neither pallor nor swollen lymph nodes were observed. There was no restriction of ocular movements. The left eye appeared normal. The parents reported that the mass had rapidly grown in the past month. The most likely diagnosis was dermoid cyst and the patient was scheduled for surgical excision. Under general anesthesia, an incision was made over the lesion (Figure 2). 

**Figure 3:**
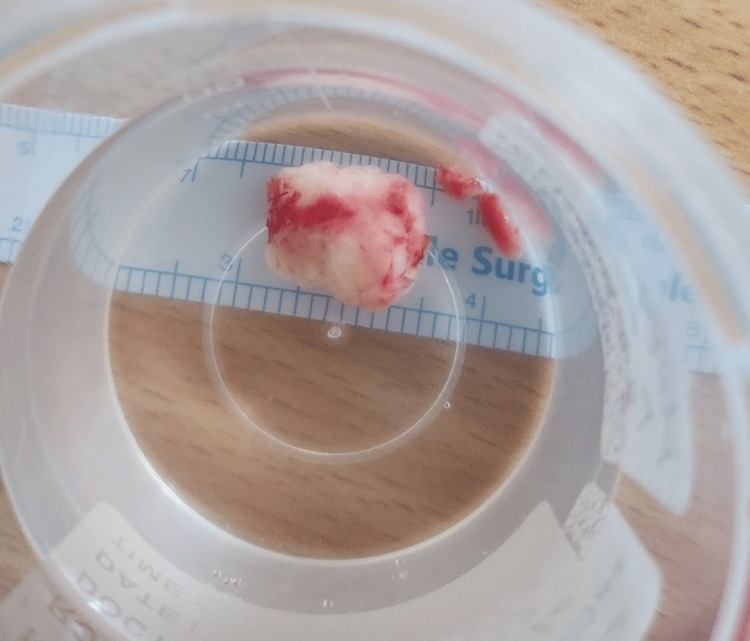
The size and appearance of the excised tumor.

After visualization of the lesion, cryopexy was used in order to detach it from the surrounding tissues without rupture (Figure 2). A high index of suspicion was raised based on the appearance of the lesion due to the presence of a feeder vessel. The mass was 1.5 cm in diameter. The incision was closed with a continuous suture and the lesion was sent for histological examination (Figure 3). 

**Figure 4:**
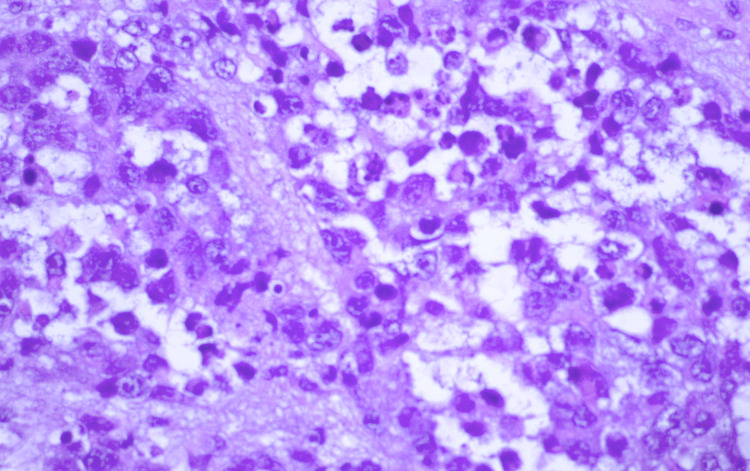
Haematoxylin and eosin stain of the tumor characterized by primitive round cells with scant cytoplasm and large hyperchromatic nuclei (magnification ×400).

The results of the excisional biopsy revealed malignant soft tissue RMS (Figure 4). 

**Figure 5:**
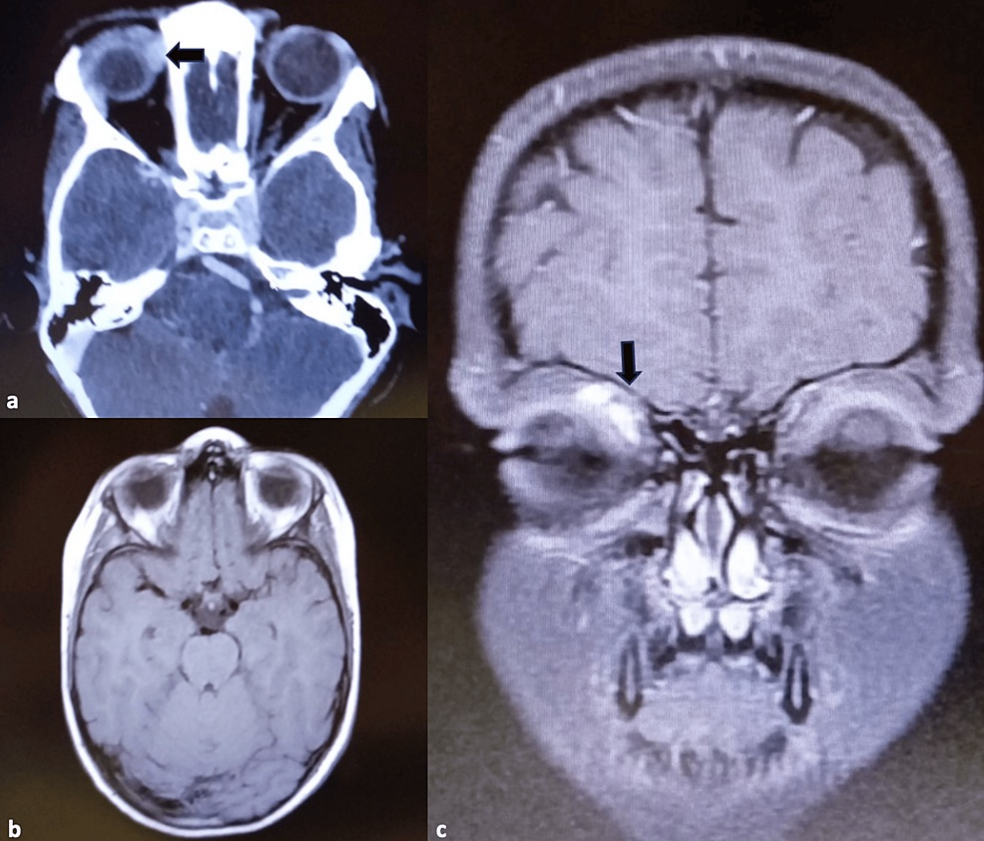
MRI of the brain and orbits. The residual tumor in the right orbit is shown with black arrows in (a) and (c). No metastasis was detected on the brain MRI (b).

The tumor tissue presented with cell clusters of various sizes, separated by variably thick fibrovascular septa, and loss of cellular cohesion in the center formed an alveolar-like appearance. Cells with rhabdomyoblastic differentiation were noticed. Mitotic figures were easily observed, and were partially atypical, with a Ki-67 index >93%. Tumor cells showed immunoreactivity for vimentin, desmin, myogenin, Myo-D1, and ALK-1. Immunohistochemical staining identified alveolar RMS type. The patient was referred to a pediatric oncologist. MRI of the brain and thorax was performed to identify the presence of metastasis. RMS was confined to the orbit and the residual tumor is shown in Figure 5. 

 The patient commenced intravenous chemotherapy with cyclophosphamide, vincristine, and actinomycin-D. 

## Discussion

RMS is the most common orbital malignancy in childhood with boys being affected more often than girls. The main sign of orbital RMS is usually a rapidly increasing unilateral proptosis, which may or may not be accompanied by globe dystopia [5,6]. RMS may also present as a lid mass. The majority of the tumors are located superonasally. In this case, the tumor was detected in the superonasal part of the orbit. Proptosis associated with signs of inflammation, chemosis, and lid edema, has also been reported as the presenting sign of RBS, mimicking orbital cellulitis [5]. The tumor may involve adjacent orbital bone, as a result of its rapid growth and aggressive nature. Intracranial involvement may take place, but metastasis in regional lymph nodes is rarely observed [5,6]. Lungs and bones are the main sites of hematogenous metastases and the prognosis for metastatic RMS is poor [2,5-8]. CT and MRI scans are useful diagnostic tools in determining the location and size of the tumor, and in evaluating the recurrence of the disease [5-7]. 

The diagnosis of RMS is verified by immunohistochemistry markers, including desmin, myogenin, Myo D1, MSA, and myoglobin [5,6]. Since vimentin and desmin may also be detected in other tumors with muscle differentiation, they are less specific [5,9]. Myogenin and Myo-D1 are transcriptional regulatory factors expressed early in skeletal muscle differentiation [5,6]. For this reason, they are more specific in diagnosing the embryonal type of RMS [6,9]. However, like vimentin and desmin, myogenin and Myo-D1 may be expressed in other tumors with skeletal muscle differentiation as well [5,6,10]. Better prognostic factors regarding survival include younger age (less than 10 years), female sex, embryonal type, and a more localized disease [5]. Orbital RMS has a favorable prognosis thanks to its anatomic location [5,6]. When possible, surgical removal of the tumor with subsequent chemotherapy/radiotherapy is suggested [6,11]. In the present case, resection of the tumor was performed and the patient received adjuvant intravenously chemotherapy. 

## Conclusions

Orbital RMS is a common primary malignant tumor in childhood, with the characteristic sign of a rapidly progressing mass. Differential diagnosis from other tumor that affect the orbit is crucial, since RMS may masquerade as various conditions, including dermoid cysts. High index of suspicion should be raised in rapidly growing lesions.
